# Determinants of health among people who use illicit drugs in the conflict-affected countries of Afghanistan, Colombia and Myanmar: a systematic review of epidemiological evidence

**DOI:** 10.1186/s13031-022-00467-9

**Published:** 2022-07-07

**Authors:** Sally O’Brien, Khine Wut Yee Kyaw, Margarita Marin Jaramillo, Bayard Roberts, Murdo Bijl, Lucy Platt

**Affiliations:** 1 Faculty of Public Health and Policy, London School of Hygiene and Tropical Medicine, London, UK; 2 Observatorio de Restitución Y Regulación de los Derechos de Propiedad Agraria, Universidad Nacional de Colombia, Bogotá, Colombia; 3 Asian Harm Reduction Network (AHRN), Yangon, Myanmar

**Keywords:** Drug use, Conflict, Drug production, Structural determinants, Displacement, HCV, HIV

## Abstract

**Background:**

Afghanistan, Colombia and Myanmar are the world’s leading heroin and cocaine producers and have also experienced prolonged periods of armed conflict. The link between armed conflict and drug markets is well established but how conflict impacts on the health and social determinants of people who use drugs is less clear. The aim was to investigate health outcomes and associated factors among people who use illicit drugs in Afghanistan, Colombia and Myanmar.

**Methods:**

We conducted a systematic review searching Medline, EMBASE, PsychINFO and Global Health databases using terms relating to Afghanistan, Colombia and Myanmar; illicit drug use (all modes of drug administration); health and influencing factors. Quality assessment was assessed with the Newcastle–Ottawa-Scale and papers were analysed narratively.

**Results:**

35 studies were included in Afghanistan (n = 15), Colombia (n = 9) and Myanmar (n = 11). Health outcomes focused predominantly on HIV, Hepatitis C (HCV), Hepatitis B and sexually transmitted infections (STIs), with one study looking at human rights violations (defined as maltreatment, abuse and gender inequality). Drug use was predominantly injection of heroin, often alongside use of amphetamines (Myanmar), cocaine and cocaine-based derivatives (Colombia). Only one study measured the effect of a period of conflict suggesting this was linked to increased reporting of symptoms of STIs and sharing of needles/syringes among people who inject drugs. Findings show high levels of external and internal migration, alongside low-income and unemployment across the samples. External displacement was linked to injecting drugs and reduced access to needle/syringe programmes in Afghanistan, while initiation into injecting abroad was associated with increased risk of HCV infection. Few studies focused on gender-based differences or recruited women. Living in more impoverished rural areas was associated with increased risk of HIV infection.

**Conclusions:**

More research is needed to understand the impact of armed-conflict and drug production on the health of people who use drugs. The immediate scale-up of harm reduction services in these countries is imperative to minimize transmission of HIV/HCV and address harms associated with amphetamine use and other linked health and social care needs that people who use drugs may face.

**Supplementary Information:**

The online version contains supplementary material available at 10.1186/s13031-022-00467-9.

## Background

People who inject drugs (PWID) can be more vulnerable to infectious diseases such as HIV, Hepatitis C (HCV), tuberculosis, poor mental health conditions, soft tissue infections and abscesses, overdose and death [1, 2]. An estimated 43% of global HCV transmission is attributed to unsafe injecting practices among PWID [3]. Approximately 10% of new HIV infections globally and over 40% outside Sub-Saharan Africa occur among PWID [4]. More broadly, people who use drugs (PWUD) (i.e. including other modes of drug administration) are at increased risk of experiencing violence, homelessness, incarceration, social and economic exclusion, and reduced access to health services. [1, 5–7] The epidemiological context as well as social and structural factors, types of drugs used and method of administration all shape variances in health outcomes among PWUD.

The ‘risk environment’ concept, developed to understand drug-related harms among PWUD, examines different types (physical, social, economic, and political) and levels of environmental influences (micro and macro), in line with broader efforts to address structural determinants of health [8, 9]. Epidemiological evidence documents the associations between macro-structural factors (laws, housing and economic insecurity, migration, education and stigma) as well as community factors (policing, drug use setting access to peer-led services) and increased risk of HIV and HCV among PWUD [10]. Criminalisation and repressive policing practices has been shown to increase risk of HIV and HCV among PWID, while mathematical modelling shows that the cessation of problematic policing practices such as physical harassment could have a substantial impact on reducing incidence of HIV infection by reducing syringe sharing and other risk behaviours [10].

Afghanistan, Colombia and Myanmar are the world’s leading drug producers, accounting for more than 90% of illiegal opium and heroin production and 50% of cocaine production globally [11]. Myanmar is also one of the main producers of amphetamine type stimulants (ATS) following a decade of increasing demand for ATS in Asian and European drug markets [12]. Evidence suggests elevated drug use in countries where drugs are produced [13]. The increase in drug use is thought to be attributable to increased availability of drugs as a result of reduced enforcement of anti-drug policies during times of armed conflict, changes in social norms during and after conflict and initiation of drug use as a coping mechanism for exposure to armed conflict and forced displacement [14]. The global prevalence of opiate use was estimated to be 1.2% in 2019. This compares to 2.6% in Afghanistan in 2015 and 0.02% for opiates and 0.6% for cocaine in Colombia in 2019. [14] While in Myanmar 0.3% of the total population (15–64 years) are estimated to inject drugs, with higher prevalence among men aged 15 or older in Waingmaw in Kachin state (2.3–4.8%) and in Muse, Shan State (2.8–4.6%) [15].

Prevalence of HIV among PWID is estimated to be 4% in Afghanistan, 35% in Myanmar and 8.4% in Colombia [15–17]. There is substantial evidence showing the effectiveness of harm reduction interventions including needle syringe programmes and opioid substitution therapy in preventing HIV and HCV transmission [18–20]. In Myanmar, needle/syringe programmes, opioid substitution therapy and the provision of ART have been scaled up over the last 10 years to prevent and treat HIV among PWID. This was facilitated by the introduction of more supportive drug policies in 2016 that sought to reduce drug production and decriminalise drug possession in recognition of the negative social and health consequences for PWUD, their families and communities due to the very high rates of incarceration for drug possession in Myanmar [21]. While the extent to which the new policy is being implemented is unclear, the policy actively endorses a harm reduction approach to drug treatment [12, 15]. In Afghanistan, policies prioritise the eradication of drugs and punishment of drug users with forced rehabilitation or treatment. Incarceration for minor drug offences and violent anti-drug policing by the police, military and paramilitary forces is common [22]. In Colombia, drug policy is less clear. Legislation in 2021 eliminated the punishment of possession for minimum quantities amounting to personal doses, but in practice repressive enforcement by the police on the street is often used. Significant barriers to harm reduction services exist across all three countries and coverage remains insufficient [23].

All three countries have suffered protracted armed conflict leading to large-scale forced displacement with millions moving within the countries as internally displaced persons (IDPs) or into neighbouring countries as refugees. The link between armed conflict and drug production in Afghanistan and Colombia is well documented, with military, paramilitary and rebel forces often funded by drug economies [24, 25]. Armed conflict and consequences such as forced displacement have been linked to elevated drug use, as well as exacerbating challenges to the implementation of interventions related to resource limitations, stigma, and low political prioritization [14, 26, 27]. However, the inter-relationship between drug production, conflict and the health of PWUD is less well described. Understanding the specific risk and protective factors for health outcomes among PWUD in the context of heightened availability of drugs and armed conflict is essential to design effective interventions, improve drug policies and reduce the burden of ill-health. We undertook a systematic review with the aim to investigate health outcomes and associated factors including armed conflict and its consequences among people who use illicit drugs in Afghanistan, Colombia and Myanmar.

## Methods

The systematic review followed PRISMA guidelines to explore the relationship between conflict, drug use and health in Afghanistan, Colombia and Myanmar [28]. The specific research questions were: (i) what does the available evidence tell us about the health of drug users? (ii) What are the factors associated with poor health outcomes? (iii) how do contextual factors influence health outcomes? (iv) What is the quality of the current evidence on the health of drug users in Afghanistan, Colombia and Myanmar?

### Eligibility criteria

The population of interest was PWUD living in, or originating from Afghanistan, Colombia or Myanmar including populations who have resettled in other low- or middle-income countries (e.g. as refugees or migrants). We excluded studies of displaced PWUD now living in high income countries. Drug use included all forms of illicit drugs, excluding alcohol and tobacco and both injecting and other modes of administration. We included all health outcomes, access to any form of health or social services and behaviours known to be associated with poor health among PWUD (e.g. condomless sex, sharing of needles/syringes or drug paraphernalia). Only primary research published after the year 2000 was included to capture studies conducted during recent periods of conflict and ceasefires in each of the three countries. Inclusion and exclusion criteria are included in Table 1.

**Table 1:** Eligibility criteria

	Inclusion criteria	Exclusion criteria
Population	People using illicit drugs (all modes of administration)	Studies on alcohol and tobacco or other legal substances Studies with family members of drug users
	People living in Afghanistan, Myanmar, Colombia	Studies of citizens from all other countries
	Afghan, Myanmar, or Colombian citizens who have settled in other LMIC countries	Studies in high-income countries Studies with Afghan, Myanmar, or Colombian citizens in LMICs that do not distinguish between them and host populations
Comparison	Factors associated with health outcomes specifically among people using drugs, including access to any type of health or social support services	Studies that do not examine factors influencing health outcomes Studies that do not provide statistical tests of significance for factors associated with health outcomes
Outcome	Any health outcome among people using illicit drugs	Studies on use of alcohol, tobacco and other legal substances Studies of health outcomes among general populations
Study type	Primary quantitative studies English or Spanish languages Studies published from 2000 onwards	Qualitative studies Quantitative studies not providing statistical tests of significance for factors associated with health outcomes Policy studies Reviews, case reports, editorial, commentaries Not in English or Spanish Published before 2000

### Search strategy

Search terms used covered three key domains: (1) countries of interest (Afghanistan, Colombia and Myanmar); AND (2) drug use; AND (3) health outcomes (HIV, hepatitis B, hepatitis C, sexually transmitted infections, mental health, violence, access to services). The full search strategy for each database can be found in Additional file 1: Appendix 1. Both key word and subject heading (MeSH) searches were used. EMBASE, Medline, PsychINFO and Global Health databases were searched via Ovid in August 2021.

### Study selection and data extraction

All study designs and all languages were considered for the initial search but only English-language and Spanish-language studies were considered at the full text review and data extraction stages. Study selection and data extraction were completed by SOB, MMJ, BR and LP. Any discrepancies between authors were resolved through joint review of the differences and agreement then reached. Citations were deduplicated and screened by title and abstract. Studies that did not report on associated risk factors were also excluded at the screening stage. Full text articles for all remaining citations were obtained to determine eligibility for inclusion in the final review. Reference lists of included articles were reviewed to identify any potential further studies. Data were extracted on: author and date of publication; location (country; city); study design; population characteristics (types of drugs used; exposure to conflict; migration or drug production); sample size; recruitment method; outcome measure; type of analysis; findings; quality appraisal score. Where both bivariable and multivariable analyses were reported, only multivariable results were extracted. If no multivariable analysis was done, bivariable results were extracted. Only results that were considered statistically significant (*p* < 0.05) were extracted.

### Analysis

Results were synthesized narratively. First, studies were grouped into categories including: health outcomes; risk behaviors; and access or utilization of health services. Second, significantly associated risk factors were extracted for health outcomes, risk behaviors, and access/use of health services. The associated risk factors were separated out into individual and structural level factors. Multivariable results are reported where available, but in their absence univariable results are presented. Individual level factors were those endogenous to the individual such as age, gender, drug use behaviours (e.g. injecting practices, sharing needles) or sexual practices. Structural level factors were anything exogenous to the individual and included: migration; city vs rural location; education; employment; prior incarceration. In this paper we highlight structural-level determinants, but individual-level factors are reported.

### Quality assessment

A quality assessment was conducted using the Newcastle-Ottowa Scale (NOS) tools for cross-sectional, cohort and case control studies and a total score was calculated for each study based on checklist items. Quality assessment focused on assessing the strengths and weaknesses of each study and no studies were excluded on the basis of the quality assessment findings.

## Results

### Study selection and characteristics

**Fig. 1:**
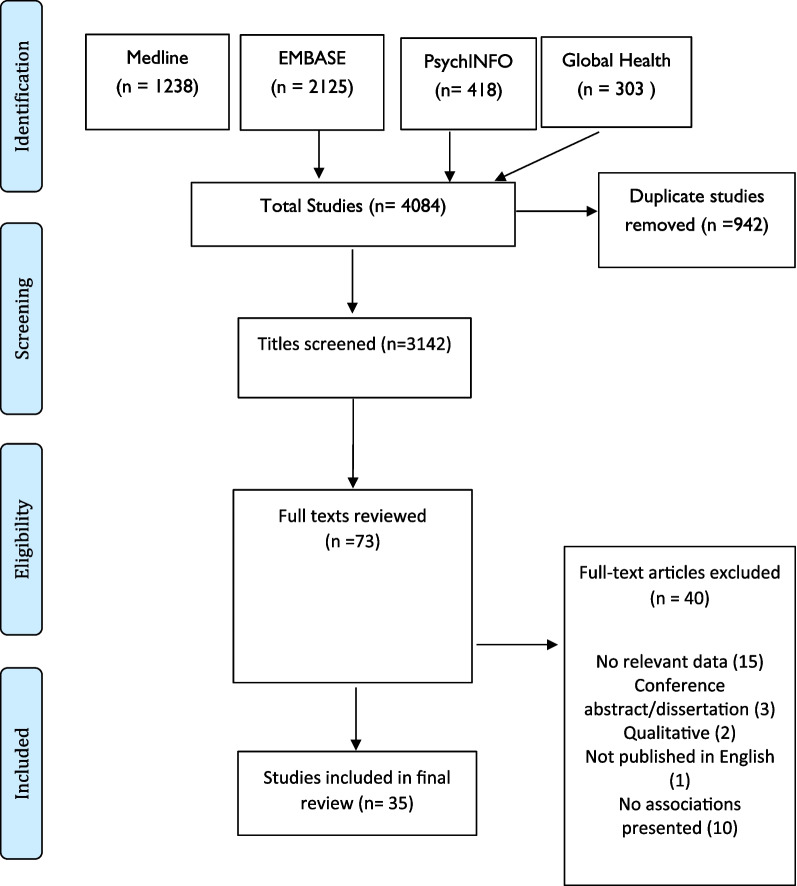
Results of the study selection process

Bibliographic database searching retrieved 4084 articles, of which 942 were duplicates. The remaining 3142 articles were screened by title and abstract and 73 full text articles were retained for review. Of these, 35 articles met the inclusion criteria and were included in the final review (Fig. 1) [29–63]. The characteristics of the final eligible studies are provided in Table 2.

**Table 2:** Characteristics of included studies

Author/ref	Study design (recruitment, location)	N	Nature of drug use	% male	Conflict/ contextual indicator	Age (mid point)	Outcomes	Quality score
*Afghanistan*								
Todd, 2007a, 2007, 2009 [53–55]	Cross-sectional (community/DTS, Kabul)	464	PWID Heroin	100% (1 F)	86.4% lived or worked outside the country in last 10 years (primarily due to conflict)	60% > = 30 years	HIV, HCV, HBV	7/10
							Lived outside Afghanistan in last 10 years	5/10
							Access to DTS	5/10
Todd, 2010 [56]	Cross-sectional (community/DTS, Hirat, Kabul, Jalalabad, Mazar-i-Sharif)	1078	PWID Heroin	100%	96.8% lived or worked outside country in last 10 years (Pakistan, Iran, other)	28	Syphilis Ever condom use with female sex worker	8/10
Todd, 2011[57]	Cross-sectional (TLS, Kabul)	483	PWID Heroin with Avil	100%	64.7% lived outside Afghanistan in last 5 years, 63.1% ever in prison	29.6	HIV, HCV, HBV Access to NSP	6/10
Bautista, 2010[31]	Cross-sectional (community/DTS, Kabul)	459	PWID Heroin	100%	N/A	30.4	HCV	6/10
Nasir, 2011 [41]	Cross-sectional (TLS. Hirat, Jalalabad, Mazar-i-Sharif)	623	PWID Heroin/Avil	100% (1 F)	85.2% lived or worked outside of Afghanistan; 62.9% ever in prison	N/A	HIV, HCV, HBV Sharing n/s; re-injecting blood; help with injecting	7/10
Abadi, 2012 [29]	Cross-sectional (DTS, n/a)	176	PWUD Opium, crystal, hashish, heroin (no injecting reported)	0% (all F)	5% forced to work in poppy cultivation; 13% lost family member to conflict in past 2 years	39	Mental health, human rights violations	2/10
Ruisenor-Escudero, 2014 [46]	Cross-sectional (RDS, Kabul, Herat, Mazar-i-Sharif)	548	PWID Heroin, opium, crystal	100%	88% had lived outside of Afghanistan; 40.1% unable to read or write	28	HIV and HCV	9/10
Ruisenor-Escudero, 2015 [45]	Cross-sectional (OST clinic, Kabul)	83	PWID Heroin	100%	51.8% ever been in prison	32.3	Retention into OST	4/10
Todd, 2015; 2016 [58, 59]	Cohort (TLS, Kabul)	385	PWID Heroin	100%	65% lived outside Afghanistan in last 5 years; 63% ever been in prison; 26% homeless; 36% initiated injecting as a refugee	28	HCV and HIV Prevalence and incidence	7/9
							Syringe sharing; paying women for sex; STI symptoms*	5/9
Rasekh, 2018 [43]	Cross-sectional (Convenience, Kabul)	327	PWUD Heroin, Crystal (94% smoking)	100%	77.4% had migrated to Kabul from other provinces	30.1	Drug treatment completion	7/10
Rasekh, 2019 [44]	Cross-sectional (DTS Kabul)	410	PWUD 13.4% inject; heroin (86%; crystal methamphetamine 7.6%)	100%	53.4% illiterate; 42.2% started using drugs in other countries (38.8% in Iran)	31.5	HCV, HIV, HBV Prevalence of injecting	6/10
*Pakistan*								
Zafar,2003[62]	Cross-sectional (DTS, (Quetta)	956	PWUD 97% Heroin but 13.2% inject	100%	14.9% from Afghanistan, 20% homeless, 36.8% ever arrested; 69% of Afghans inject	35	Currently injects drugs, use of opiates as first drug; sex with sex worker	4/10
*Colombia*								
Berbesi-Fernandez, 2013 Saluld mental[34]	Cross-sectional (RDS, Pereira, Medellin)	540	PWID Heroin (100%) basuco (40.7%) cocaine (60%)	92.8%	76.6% low socio-economic status	85.7% < 30	Needle/syringe sharing	3/10
Berbesi-Fernandez, 2013 JSU[33]	Cross-sectional (RDS, 3 cities n/a)	796	PWID Heroin (100%) Cocaine (58.4%)	92%	62.5% low income status	26.6	Needle/syringe sharing	6/10
Berbesi-Fernandez, 2015 [36]	Cross-sectional (RDS, Armenia)	250	PWID Heroin	87%	20.8% street vendors, 83% low-level socio-economic status	26.8	HCV and HIV	6/10
Berbesi-Fernandez, 2017[37]	Cross-sectional (RDS, Armenia, Cucuta, Medellin and Bogota)	668	PWID Heroin	82.2%	n/a	26	HCV and HIV	6/10
Toro-Tobon 2018[61]	Cross-sectional (RDS, Armenia, Bogotá, Cúcuta and Pereira)	918	PWID Mainly heroin	86%	75.% % low socioeconomic level	26	HCV	7/10
Toro-Tobon 2020 Berbesi-Fernandez, 2017 [37, 60]	Colombia Armenia, Bogotá, Cúcuta and Pereira)	1123	PWID Heroin and cocaine (% n/a)	86.3%	8.4% engaged in illicit work 59.3% engaged in informal work	52.1% < 25	HCV, HIV coinfection	7/10
							Sharing needles/syringes	7/10
Berbesi-Fernandez, 2020 [35]	Cross-sectional (RDS, Medellin)	224	PWID (not stated)	86.2%	80.6% less than minimum wage 63.4% sold drugs		HIV	7/10
Borda, 2021 [38]	Cross-sectional (DTS, Armenia, Pereira, Cali, Medellin)	171	PWID Heroin (87.1%) basuco (51.5%), cocaine 31%)	84.8%	26.7% homeless; 41.5% unemployed	29.7	HIV, HV prevalence, testing and treatment	3/10
*Myanmar*								
Morineau, 2000[40]	Cross-sectional (DTS, Myktyina)	272	PWUD Heroin, opium 46.7% inject	98%	n/a	49.3%16–25 years	Sharing injecting equipment	2/10
Swe, 2010 [51]	Cross-sectional (DTS, Shan State)	217	PWID Heroin, opium	97.2%	15.7% illiterate, 51.6% rural locations	32.8	HIV	7/9
Swe, 2012 [52]	Cross-sectional (DTS, Shan, Kachin, Mandalay, Yangon)	590	PWID Heroin/opium. (89% injecting)	98%	17.6% unemployed	10% < 21 years	HIV	2/10
Saw, 2013; 2016[49, 50]	Cross-sectional (RDS, Lashio)	368	Heroin (PWID) Stimulants (58.6%) and heroin (41.4%) (PWUD)	100%	12.7% PWID and 31.9% PWUD internal migrants	29.8 PWID; 25.5 PWUD	Ever testing for HIV	7/10
		210	PWUD Stimulants (58.6%) and heroin (41.4%)	100%	31.9% internal migrants 16.2% non-regular employment	25.5	Exchange sex	8/10
Saw, 2014 [47]	Cross-sectional (RDS, Muse)	776	PWUD Methamphetamine	58.6%	48.8% internal migrants; 41.9% unemployed	21.2	Ever testing for HIV	7/10
Saw, 2018[48]	Cross-sectional (RDS, Muse)	1183	PWUD Methamphetamine	65.2%	61.4% internal migrants 70.8% unemployed	24.5% under 20	Sexual risk (inconsistent condom use; 2 or more sex partners in last 5 months, history of STI or current infection)	7/10
O’Keefe, 2018[42]	Cross-sectional (convenience and snowball, Yangon, Mandalay, Pyin Oo Lwin)	513	PWID Heroin	97%	25% unemployed, 4% unstably housed	27	Coverage of NSP	7/10
Aye, 2018[30]	Cohort (DTS, Yangon)	642	PWID Heroin	97.7%	n/a	27	HIV, HBV, HCV Drop out of OST	8/9
Lum, 2020[39]	Cohort study (DTS, Mykitkyina)	287	PWID Heroin	100% (1 F)	n/a	28	ART initiation	6/9
*Myanmar/China border*								
Zhou, 2011 [63]	Cross-sectional/ Myanmar: community China: community & OST	721	PWID Heroin		403 Chinese/318 Burmese)	32.3 (Chinese) 31.8 (Burmese)	HCV, HBV, HIV	4/10

*F* Female; *M* Male *N/A* not available; *TLS* Time location sampling; *RDS* Respondent Driven Sampling; *DTS* Drug Treatment Service; *PWID* People who inject drugs; *PWUD* People who use drugs; *ART* anti-retroviral therapy *OST* Opioid Substitution Therapy; *NSP* Needle Syringe Programme

^*^STI symptoms defined as dysuria, penile discharge and/or genital ulcers or warts

The included studies were published from 2000 to 13th August 2021. Fourteen studies were conducted in Afghanistan [29, 31, 41, 43–46, 53–59], one study was with Afghan refugees in Pakistan [62], nine were in Colombia [32–38, 60, 61], ten were in Myanmar[30, 39, 40, 42, 47–52] and one was in China and Myanmar [63]. Thirty were cross-sectional in design, four were cohort studies and one case–control study.

Most studies focused on PWID (n = 26) and primarily heroin injection. All studies in Colombia were among PWID, primarily heroin and also cocaine or cocaine derivatives. Five studies in Myanmar focused on injecting heroin and non-injecting of methamphetamines and four studies in Afghanistan focused on non-injection of opioids. Between 64 and 97% of PWID in Afghanistan had lived outside the country in the last 5–10 years during periods of conflict. One study stated that moving abroad was a result of conflict and between 36 and 42% reported initiating drugs while away [41, 43, 44, 46, 53–59]. A study in Afghanistan reported that 5% of women in a drug treatment centre were forced to work in poppy cultivation, 19% were forced to use drugs and 13% had lost a close family member due to conflict in the last two years [29]. Between 32 and 77% of samples in Myanmar and Afghanistan were internal migrants [43, 47–50, 63]. There was limited demographic characteristics reported among the Colombian studies, the majority of the samples were from urban centres, reported to be of low socio-economic levels. A study in Medellin, a city in Colombia, reported that 63.4% of the sample sold drugs [35]. Between 50 and 60% of Afghanistan populations reported a history of imprisonment [41, 45, 57] between 4 and 20% were homeless or unstably housed across the three countries[38, 42, 59, 62] and between 15 and 52.4% were unable to read or write in Myanmar and Afghanistan [44, 46, 51]. In all studies except one, the majority of the sample were male (82–100%). In Afghanistan one study focused on women only and two studies in Myanmar included both men and women. All these studies focused on non-injecting drug use [29, 47, 48]. There was little information reported on ethnic identities. Studies from Shan State in Myanmar were characterised predominantly by Shan, Kachin, Burma ethnic groups but also included people identifying as Kayar, Kayin, Chin Mon, Rakhine, multi-ethnicity, aggregated into one group due to small numbers. [47–52] One study across three undisclosed treatment sites in Afghanistan included people identifying as Pashtun, Turkmen, Tajik and Hazara ethnic groups [29].

### Quality assessment

Among the thirty cross-sectional studies, just under half described sample size calculations and none described non-respondents. Representativeness of samples is difficult to ascertain among populations without a priori-sampling frames. Twelve studies used respondent-driven sampling to account for sampling biases, whilst the others used either purposive, convenience, community-based recruitment, or a variation on time-location sampling. All but two of the cross-sectional studies adjusted for at least one confounding factor and presented p-values and confidence intervals for significant associations. The quality of the four cohort studies was generally low with the most common weaknesses being unclear definitions of the exposed/unexposed groups and high loss-to-follow-up. The overall summary scores for each study are given in Table 2 and the detailed results for each study are provided in Additional file 1: Appendix 2.

### Health outcomes

Nineteen studies investigated physical health outcomes (Table 3). Twelve of these studies measured prevalence of HIV, ranging between 0.2% to 7.1% in Afghanistan, [41, 44, 46, 54, 57]15% to 27% in Myanmar [30, 51, 52, 63], and 2.6% to 5.3% in Colombia [35–37, 60]. Twelve studies reported HCV prevalence ranging between 11% to 40.3% in Afghanistan [44, 46, 54, 57], 48.1% to 76% in Myanmar, [30, 63] and 17.5% to 37% in Colombia [31, 35–37, 60, 61]. Six studies reported HBV prevalence and this varied from 3.7% to 5.8% in Afghanistan [41, 44, 54, 57] and from 4% to 43.1% in Myanmar [30, 63]. Prevalence of syphilis was 1.2% and 3.72% in two studies in Afghanistan [56, 57]. Incidence of HIV was 1.5 per 100 person years and incidence of HCV was 35.6 per 100 person years in Afghanistan [59]. One study also reported on STI symptoms [58].

**Table 3:** Health outcomes and associated individual and structural level risk factors

Author/ref	Outcome/ prevalence	Analysis	Findings on associated factors
*Afghanistan*			
Abadi, 2012 [29]	Human rights violation defined as maltreatment, abuse, gender inequality: Maltreatment (threatened/denial of food or shelter; forced social isolation, drug use or working in poppy cultivation) = 36% Abuse (physical or sexual assault) = 35% Gender inequality (denied education, driving a car or being alone in public) = 4% Suicidal ideation = 41%; attempted suicide within 30 days of entering drug treatment centre = 27% Social function (physical/emotional health limits social activities) = 91%	MV	*Associated with any human rights violations (maltreatment, abuse or gender inequality)*: being married AOR 5.08; Pashtun ethnicity AOR 5.80; literate AOR 14.7; unemployed AOR 4.76; entering drug treatment because of their own desire AOR 2.30; lower levels of social functioning AOR 1.72; previous suicide attempt AOR 3.99. All significant at *p* < 0.05 level *Factors associated with social functioning*: Maltreatment (AOR = 2.36), physical/sexual abuse (AOR = 12.24), any human rights violation(AOR = 1.72). *p* values not reported *Factors association with suicide attempt*: Any human right violation (AOR = 2.80), maltreatment (AOR = 5.74), sexual/physical abuse (AOR = 26.05) and one human right violation (AOR = 3.99). *P* values not reported *Factors associated with suicide ideation*: None significant
Bautista, 2010[31]	HCV: 37%	MV	*Associated with HCV among younger injectors*: ever-sharing needles AOR 7.8 (95%CI 3.6–16.8); unemployment AOR 2.8 (95% CI 1.1–7.3) *Associated with HCV among older injectors*: heroin use AOR 3.0 (95%CI 1.0–8.7); ever-sharing needles AOR 3.4 (95% CI 1.7–7.0); each additional year of injecting AOR 4.45, 95% CI: 2.92–7.66)
Nasir, 2011[41]	HIV: 1.8% (95% CI: 0.88–3.2) HCV: 36.0% (95% CI 33–41) HBV: 5.8% (95% CI 3.9–7.6)	MV	*Associated with HBV:* higher monthly income AOR 1.74 (95%CI 1.55–1.96); paying a sex worker for sex in the last 6 months AOR 2.24 (95%CI 1.64–3.06); ever having sex with another male AOR 0.61 (95%CI 0.36–1.02) *Associated with HCV*: sharing injecting equipment last 6 months AOR 1.83 (95%CI 1.25–2.69); ever incarcerated AOR 1.79 (95% CI 1.16–2.77); ever had sex with man/boy AOR 0.69 (95% CI 0.58–0.82); median income > 3800 Afs AOR 0.76 (95%CI 0.60–0.97) *HIV:* No statistically significant correlates
Rasekh, 2019[44]	HIV 0.2% HBV 3.7 HCV 11% HIV/HCV 0.2% HCV/HBV 0.5%	MV	*Associated with HCV:* shared needles and use of drugs by injection AOR 5.40 (95% CI 2.60–11.23) *Associated with any viral infection:* use of drugs by injection AOR 3.57 (95% CI 1.76–7.24)
Ruisenor-Escudero, 2014[46]	HIV: 7.1% HCV: 40.3%	MV	*Associated with HIV:* living in Herat AOR 15.2 (95%CI 1.5–145.2); prior incarceration AOR 9.4 (95%CI 1.1–78.0); injecting drugs for > 3 years AOR 7.2 (95%CI 1.3–39.6); being positive for HCV AOR 15.7 (95%CI 3.4–72.5) *Associated with HCV:* living in Kabul AOR 1.8 (95% CI1.0–3.2); living in Herat AOR 1.9 (95%CI 1.0–3.8); injecting for 1–3 yrs AOR 2.3 (95%CI 1.5–3.7); injecting for > 3 years AOR 5.4 (95%CI 3.0–9.5); being positive for HIV AOR 14.9 (95%CI 3.2–70.2)
Todd, 2007[54]	HIV: 3.5% HCV: 36.6% HBsAg 6.5%	MV	*Associated with HIV*: None *Associated with HBsAg*: injecting drugs in prison (OR 3.23 95% CI 1.16–9.00) *Associated with HCV*: ever N/S sharing (OR 2.60 95% CI 1.71–3.96); being married (OR = 0.60 95% CI 0.40–0.92); higher educational level (OR 0.51 95% CI 0.29–0.88); duration injection (> 3 years) (Or = 3.28 95% CI 2.17–4.96); injections by a nonmedical provider (OR = 2.71 95% CI 1.26–5.82)
Todd, 2010 [56]	Syphilis 3.72% (95% CI 2.66%-5.06%)	MV	*Associated with syphilis*: any other STI diagnosis (adjusted odds ratio AOR 3.84 (95%CI 1.12–13.19); paying a sex worker for sex AOR 3.82 in the last 6 months (95%CI 1.23–11.85); < 6 years formal education AOR 2.20 (95%CI 1.04–4.68)
Todd, 2011[57]	HIV: 2.1% (95%CI: 1.0–3.8) HCV Ab: 36.1% (95%CI: 31.8–40.4) HBV: 4.6% (95%CI 2.9–6.9) syphilis: 1.2% (95%CI 0.5–2.7)	MV	*Associated with HIV*: ever share needles or syringes AOR 5.96 (95%CI 1.58–22.38) *Associated with HBV:* Current needle and syringe program use AOR 0.36 (95%CI 0.14–0.94) *Associated with HCV*: ever have abscess at injecting site AOR 2.22 (95%CI 1.33–3.70); ever share needles or syringes AOR 2.33 (95%CI 1.38–3.95); initiated injecting outside Afghanistan AOR 1.95 (95%CI 1.26–3.04); frequency daily injections (# injections) AOR 1.47 (95%CI 1.11–1.94); duration of injecting (per year) AOR 1.05 (95% CI 1.00–1.10); age (per year) AOR 1.04 (95% CI 1.01–1.07)
Todd, 2015[59]	HCV incidence: 35.6/100 p-y (95%CI 28.3–44.6) HIV incidence: 1.5/100 p-y (95%CI 0.6–3.3)	MV	*Associated with HCV*: changing from injecting to smoking adjusted hazard ratio (AHR) 0.53 (95%CI 0.31–0.92) *Associated with HIV*: duration of injecting drug use AHR 1.09 (95%CI 1.01–1.18)/year; sharing needles/syringes AHR 10.08 (95% CI 1.01–100.3) No statistically significant association between conflict and HIV/HCV
Todd, 2016[58]	STI symptoms Sharing of needle/syringes Paying women for sex	MV	*Associated with STI symptoms*: Impact of conflict AOR 1.98 (95% CI 1.10–3.56) *Associated with needle/syringe sharing*: Impact of conflict AOR 6.23 (95% CI 1.41–27.6) Conflict defined as enumeration of of anti-government attacks in KabulProvince between February and May 2009 that resulted in dsplacement throughout the city
*Colombia*			
Berbesi-Fernandez, 2015 [36]	HCV: 31.0% antibodies; 22.3% active infection HIV:2.6% (plus 1.1% were undetermined)	MV	*Associated with HCV*: People who did not got syringes at drugstores in the last six months AOR 2.7 (95% CI 1.32–5.48)
Berbesi-Fernandez, 2017[37]	HCV: 17.5% HIV:4.2% HIV/HCV coinfection 54%	MV	*Associated with HCV*: Having HIV AOR 6.87 (95%CI 2.86–16.06); injection with people with hepatitis AOR 2.45 (95%CI 1.33–4.53); shared syringes AOR 1.9 (95%CI 1.12–3.2)
Berbesi-Fernandez, 2020[35]	HIV- 3.6%	UV	Association with being HIV + . More than three people with whom a needle was shared (reference category of none): OR 5.07 (CI 1.19–21.55), *P* 0.03; always injected with used needles accompanied by a close friend in last six months (ref category of almost always): OR 10.69 (95% CI 2.26–50.61), *p* < 0.01). Nothing significant in multivariable analysis
Toro-Tobon. 2018[61]	HCV: 27.3%	MV	*Associated with HCV*: In Pereira and Armenia, high injection frequency AOR 2.0 (95% CI 1.1–3.6) and AOR 2.5 (95% CI 1.40–4.20), respectively; increased frequency of using gifted, sold, or rented needles or syringes AOR 4.5 (95% CI 1.00–7.10) in Pereira; HIV seropositivity AOR 16.9 (95% CI 3.51–81.52) in Cúcuta
Toro-Tobon 2020[60]	HIV 5.3% HCV 28.9% HIV/HCV co-infection 3.3%	UV	*Associated with HIV/HCV co-infection*: female sex OR 2.2 (95% CI 1.0–4.7) *Associated with HIV OR HCV mono-infection*: higher education protective OR: 0.6 (95%CI 0.4–0.8) *Associated with co-infection*: injecting ≥ 4 times/day OR 3.5 (95%CI 1.7–7.2); cleaning needles and syringes with water OR 3.2 95%CI 1.6–6.3); passing drug mix between syringes OR: 2.5 (95%CI 1.3–5.3); injecting in illicit indoor shooting galleries OR 2.4 (95%CI 1.0–5.3); being injected by someone who charges for injecting OR 2.3 (95%CI 1.0–5.2)
*Myanmar*			
Swe, 2010[51]	N/A	UV	*Associated with HIV*: being illiterate OR 2.31 (95%CI 1.09–4.83); living in a rural location OR 2.42; 95% CI 1.36–4.29); using a used syringe (vs disposable) at first injection OR 5.13 (95%CI 2.79–9.44); sharing syringes at first injection OR 4.50 (95%CI 2.49–8.16); returning a used syringe OR 3.32 (95%CI 1.01–6.86); having had drug treatment OR 4.91 (95%CI 1.84–13.14)
Swe, 2012 [52]	HIV: 25.8%	UV	*Associated with HIV*: being female OR 5.96 (95%CI 1.31–30.45); using a ‘used’ syringe at first injection OR 1,81 (95%CI 1.23–2.68); sharing syringe at first injection OR 2.98 (95%CI 2.00–4.44)
Aye, 2018[30]	HIV: 15–17% HBV: 4–7% HCV: 68–76%	MV	*Associated with HIV*: Age (reference group aged 21–30 years): 30–40 yr AOR 1.7 (95%CI 1.1–2.7); > 40 yr AOR 2.1 (95% CI 1.2–3.6) *Associated with HCV*: Being single AOR 1.2 (95%CI 1.1–1.3)
*Myanmar and China*			
Zhou, 2011[63]	Prevalence (Burmese): HCV: 48.1%. HBV: 43.1%. HIV: 27.0% Prevalence (Chinese) HCV: 69.0% HBV:51.6%; HIV 33.7%	UV	*Associated with all infections*: more prevalent among Chinese PWID compared to Burmese PWID: HCV (69.0% vs. 48.1%, *p* < 0.001); HBV (51.6% vs. 43.1%, *P* < 0.05); HIV (33.7% vs. 27.0%, *p* > 0.05)

*MV* Multivariable; *UV* Univariable; *AHR* Adjusted hazard ratio; *AOR* adjusted odd ratio; *OR* Odds ratio

One study in Afghanistan measured human rights violation among women who use drugs and the study found that 26% had experience physical or sexual assault, 4% had been denied education or were not allowed to be alone in public and 36% had experience maltreatment (denial of food or shelter, forced drug use or working in poppy cultivation). High levels of suicide ideation (41%), recent suicide attempt (27%) and poor social functioning (91%, defined as physical/emotional health limiting social activities) were reported [29].

Key individual-level determinants of HIV or HCV infection included age, gender, injecting and sexual risk behaviours. Older age was associated with HIV among PWID in Myanmar, and with HCV in Afghanistan [30, 57]. There was some evidence of increased odds of HIV infection among women who inject drugs in Myanmar and for HIV/HCV co-infection in Colombia [52, 60]. Injecting risk behaviours including injecting with used needles/syringes were associated with increased odds of HIV infection in Myanmar [51, 52], with HIV or HCV infection in Afghanistan [41, 44, 54, 57] and Colombia [32, 35]. Among samples of PWUD, injecting was associated with increased risk of any viral infection (AOR 3.57 95% CI 1.76–7.24) and changing from injecting to smoking associated with reduced risk of HCV acquisition (Adjusted hazard ratio (AHR) 0.58 95% CI 0.31–0.92) in Afghanistan [44, 59]. Increased duration of injection was associated with increased risk of both HIV and HCV in Colombia and Myanmar [31, 46, 54, 57, 59]. There was some evidence that paying for sex was associated with increased odds of HBV infection and syphilis, while having sex with another man associated with reduced odds of HBV and HCV among men who inject drugs [41, 56].

Among structural factors, there was evidence that location was associated with increased risk of HIV or HCV. PWID living in the city of Herat had higher odds of HIV (AOR 15.2 95% CI 1.5–145.2) compared with those in the city of Mazar-i-Sharif; but odds of HCV was higher among PWID in Kabul (AOR 1.8 95% CI 1.0–3.2) and Herat (AOR 1.9 95% CI 1.0–3.8). The authors attribute this to forced repatriation of Afghans between 2007 and 2008 particularly those held in prison as well as high regional mobility and the presence of drug trafficking routes in Herat. [46] Odds of HCV were higher among those who initiated injecting outside of Afghanistan (AOR 1.95 95% CI 1.26–3.04) [57]. In Myanmar, living in a rural location increased the risk of HIV among PWID (AOR 2.42 95% CI 1.36–4.29) [51]. Among PWID living in border towns in China and Myanmar near to one of the largest drug production and distribution centres in the golden triangle, PWID in Myanmar areas had lower prevalence of HIV, HBV, HCV and co-infections relative to neighbouring Chinese areas. The authors attribute this to increase injecting among PWID in China as well more sharing of needles/syringes and less well-developed harm reduction programmes [63].

Among PWID in Afghanistan prior incarceration was associated with increased odds of HCV infection (AOR = 1.79 95% CI 1.16–2.77) and some evidence of an association with HIV infection (AOR 9.4 95% CI 1.1–78.0) [41, 46]. Unemployment was a key determinant of HCV in Afghanistan (AOR 2.8 95% CI 1.1–7.3) and higher income was associated with HBV (AOR 1.74 95% CI 1.55–1.96) [31, 41]. Three studies reported associations between education and risk of HCV/HIV/HCV and syphilis. In Afghanistan there was reduced odds of HCV associated with higher education level (AOR 0.51 95% CI 0.29–0.88) and higher odds of syphilis associated with fewer years of education (AOR 2.20 95% 1.04–4.68) [54, 56]. In Colombia reduced odds of HIV or HCV mono-infection among PWID was associated with higher levels of education in Colombia (AOR = 0.6 95% CI 0.4–0.8) [60]. In Myanmar illiteracy was associated with higher odds of HIV among PWID (AOR 2.31 95% CI 1.09–4.83) [51]. Illiteracy was also associated with human rights violation in Afghanistan, defined as maltreatment, physical or sexual abuse or gender inequality (AOR = 14.74) as was unemployment (AOR 4.76), lower levels of social functioning (AOR = 1.72) and Pashtun ethnicity (AOR = 5.80) [29]. This study showed clear relationships between poor mental health and human rights violations, with all measures of human rights violations, both individual and cumulative, associated with increased odds of suicide attempt among women who use drugs [29]. One study from Afghanistan examined the impact of an intense period of conflict on the incidence of HIV and HCV but found no associations [59].

### Drug use, sexual risk behaviours and returning refugee status

Twelve studies reported associations between drug use or sexual risk behaviours. One study reported on associations between drug use behaviours and being a refugee [55]. Sharing needles/syringes was the most commonly reported injecting risk behaviour and ranged from 8 to 61% across all three countries [32–34, 58]. Three studies reported on prevalence of injecting or transitions from injecting to smoking [40, 41, 44, 62]. Sexual risk behaviours focused on condomless sex [48, 53], engaging in sex work [48] or sex with a sex worker [62].

Key individual-level determinants of sharing needle/syringes included condomless sex with a non-regular sex partner, living alone and use of cocaine or other cocaine-based derivatives alongside heroin [32–34]. Use of amphetamines was associated with engaging in sex work among men who use drugs in Lashio in Myanmar[49]; while using 2 or more types of amphetamines was associated with a composite measure of sexual risk (defined as condomless sex; 2 or more sexual partners and history of STI) among a sample of men in Muse in Myanmar [48]. Men who inject had reduced odds of sharing needles/syringes than women among a sample of PWID in Colombia (AOR 0.49 95% CI0.32–0.74) [32].

At the structural level, both internal and external migration was a key determinant of drug use and sexual risk behaviours. In Afghanistan, time spent outside the country in the past 10 years was associated with increased odds of using a condom with a female sex worker in Afghanistan (AOR 5.52 95% CI 1.83–16.71) but people who had lived in another country were less likely to report using a new needle with each injection (AOR 0.51 95% CI 0.21–0.88) [55]. Initiating drug use in another country was associated with increased odds of injecting drug use (AOR 7.46 95% CI 1.99–28.03) as was history of being in prison (AOR 3.57 95% CI 1.85–6.86) [44]. In Quetta, Pakistan, there was some evidence that people from Afghanistan were more likely to use opiates as a first drug than people from Pakistan but this was borderline significant (AOR 1.97 95% CI 0.97–4.0), and Afghani people were less likely to have sex with a sex worker (AOR 0.61 95% CI 0.37–0.99). However Afghani people had higher levels of homelessness and low income. [62] In Myanmar, originating from the city of Myitkyina was associated with increased odds of injecting compared to originating from outside Myitkyina (OR 2.4 95% CI 1.4–4.0) [40]. Internal migration was associated with increased sexual risk behaviors among women using drugs in Muse [48].

Employment and income levels were identified as determinants of drug use and sexual risk behaviours. Being employed or having a higher income was associated with increasing use of condoms with sex workers in Afghanistan, but also increased risky sexual behaviours (condomless sex, multiple partners, history of STI) among both women and men who use drugs in Muse in Myanmar [48, 56]. In Lashio in Myanmar there was some evidence that having a full-time job was associated with engaging in sex work among a sample of heroin and amphetamine users (AOR 5.10 95% CI 1.65–15.72). The authors attribute this to the ready availability of stimulants leading to people being paid for sex in drugs [49]. In Myitkyina in Myanmar, increased odds of sharing injecting equipment was associated with being a farmer (AOR 3.6 95% CI 95% CI 1.4–9.7) or a driver (AOR 3.5 95%] 1.1–12.4) compared to being a vendor or craftsman. This was attributed to the widespread availability of heroin in rural areas and reduced availability of opium traditionally smoked by farmers [40]. In Afghanistan being unemployed was associated with increased odds of injecting among people using opiates in Kabul [44]. Only one study investigated the impact of conflict in Afghanistan and reported that periods of conflict were associated with higher odds of needle/syringe sharing (AOR 6.23 95% CI 1.41–27.6) compared to peace-time [58]. These findings are summarized in Table 4.

**Table 4:** Risk behaviours (injecting, sexual and displacement) and associated individual and structural level risk factors

Author/ref	Outcome/prevalence	Analysis	Factors associated with risk behaviours
*Afghanistan*			
Todd, 2007[55]	86.4% lived outside Afghanistan in last 10 years	UV	*Living outside of Afghanistan*: always use a new needle with every injection OR 0.51 (95%CI 0.21–0.88);
Todd, 2010[56]	26.9% ever use condoms with a female sex worker	UV	*Associated with condom use with female sex worker*: living outside Afghanistan in the last decade AOR 5.52 (95%CI 1.83–16.71); higher income AOR 2.03 (95% CI 1.17–3.51); > lifetime partners AOR 1.80 (95% CI 1.32–2.45); younger age AOR 0.985 (95%CI 0.973–0.998; *p* 0.024). Adjusted for site
Nasir, 2011 [41]	Changing from smoking to injection	MV	*Associations with non-transition to injection*: Sharing needles or syringes in the last 6 months (AOR = 0.50, 95% CI 0.27–0.94), aspirating and re-injecting blood (AOR = 0.39, 95% CI 0.23–0.68), and receiving assistance with injecting (AOR = 0.36, 95% CI 0.21–0.62). Analyses controlled for the Jalalabad site (having smaller number [60.6%] of PWID changing from smoking to injection)
Todd, 2016[58]	8% Injected with used needles/syringes in past 3 months;	MV	*Associated with sharing of needles/syringes*: impacted by conflict AOR 6.23 (95%CI 1.41–27.6)
Rasekh, 2019[44]	13.4% injecting drugs	MV	*Associated with injecting drug use*: unemployed AOR 2.92 (95% CI 1.20–7.11); starting drug use in other countries AOR 7.46 (95% CI 1.99–28.03); previously in prison AOR 3.57 (95% CI 1.85–6.86)
Zafar,2003[62]	8.3% used an opiate as first drug 51.3% ever had sex with a sex worker 60% currently injecting drugs	MV	*Associated with opiate used as first drug*: Afghani vs Pakistani drug users AOR 1.97 (95%CI 0.97–4.00) *Associated with ever had sex with a sex worker*: Afghani versus Pakistani drug users AOR 0.61 (95%CI 0.37–0.99) *Associated with currently injecting drug use*: Afghani vs Pakistani drug users AOR 0.66 (95% CI 0.18–2.44)
*Colombia*			
Berbesi-Fernandez, 2013 Salud mental[34]	44% injected with a used needle/syringe in last 6 months	UV	*Associated with needle/syringe sharing*: lives alone OR 1.67(95% CI 1.0–2.3); consumption of Basuco OR 1.58 95% CI 1.11–2.23
Berbesi-Fernandez, 2013 [33]	47% of PWID used syringes received from others < 6 months	MV	*Associated with syringe sharing*: No Condon use with non-regular partner AOR 4.46 (95% CI 1.23–16.05)
Berbesi-Fernandez, 2017[32]	40.3% shared syringes	MV	*Associated with sharing needles/syringes*: male AOR 0.492 (95% CI 0.325–0.742); completed secondary education AOR 1.933 (95% CI1.324–2.874); exchanging other equipment AOR 5.553 (95% CI 4.162–7.409); crack consumption AOR 1.591 (95% CI 1.173–2.156); HCV positive AOR 1.476 (95% CI 1.076–2.024)
*Myanmar*			
Morineau, 2000[40]	61% sharing drug injecting equipment; 46.7% injecting drug use =	UV	*Associated with sharing drug injection equipment*: being a farmer (OR 3.6; 95% CI 1.4–9.7); being a driver (OR 3.5; 95% CI 1.1–12.4) compared to sellers and craftsmen (1.0); having > 1 previous detox episode (OR 1.7; 95% CI 1–3.1); exclusively intravenous drug use (OR 2.3 (95%CI 1–5.6) *Associated with an intravenous pattern of drug use*: being from Myitkyina (OR 2.4, 95% CI 1.4–4.0); drug use > 1 year (OR 3; 95% CI 1.6–5.4)
Saw, 2016 [49]	*Sex-trading*: involvement in the sex trade during the last 3 months: 40%	MV	*Associated with sex-trading*: having a regular job AOR 5.10 (95%CI 1.65–15.72); > 2 partners AOR 3.88 (95%CI 1.55–9.72; having homosexual preferences AOR 4.90 (95%CI 1.61–14.95); stimulant drug use AOR 2.38 (95%CI 1.10–5.15); using drugs ≥ twice per day AOR 2.62 (95%CI 1.19–5.77); drug use before/during sex in past 3 months AOR 2.76 (95%CI 1.08–7.03)
Saw, 2018 [48]	*Risky sexual behaviours*: Inconsistent condom use: males = 90.7%, females = 85.2% Multiple sexual partners: males = 94.2%, females = 47.2% History of STIs: males = 55.7%, females = 56.0%	MV	*Associated with engaging in risky sexual behaviours:* **Men** being employed AOR 1.42 (95%CI 1.08–1.87); using MA before /during sex AOR 1.67 (95%CI 1.23–2.28); visiting sex workers within 6 months AOR 1.41(95%CI 1.08–1.83); using > 2 ATS types AOR 1.77(95%CI 1.30–2.41) **Women** being employed AOR 1.57 (95%CI 1.13–2.18); migrated from elsewhere in Myanmar AOR 2.70 (95% CI 1.86–3.39); using MA before/during sex AOR 3.39 (95% CI 2.51–4.56); using MA once a week AOR 2.06 (95% CI 1.41–3.02); using MA > 4 times a week AOR 2.44 (95% CI 1.66–3.60); higher education (high school/ higher level of education) AOR 0.42 (95%CI 0.31–0.56) were less likely to engage in high-risk sexual behaviors compared to those with secondary or below level of education

*MV* Multivariable; *UV* Univariable; *AHR* Adjusted hazard ratio; *AOR* adjusted odd ratio; *OR* Odds ratio

### Harm reduction, drug treatment and HIV/HCV testing and treatment

Table 5 summarizes findings from ten studies that measured associations between drug treatment services or HIV/HCV testing and treatment. Two studies in Afghanistan looked at factors associated with completion or attendance at abstinence-based drug treatment centres [43, 53]. Three studies in Myanmar and Afghanistan looked at retention into opioid substitution therapy and two focused on receipt of needle/syringes at harm reduction programmes [30, 42, 43, 45, 57]. One study in Colombia and three in Myanmar focused on use of HIV testing and treatment [38, 39, 47, 50].

**Table 5:** Use of harm reduction, drug treatment, HIV/HCV testing and treatment services and associated individual and structural level risk factors

Author/ref	Outcome/ prevalence	Analysis	Findings on associated factors
*Afghanistan*			
Todd, 2009 [53]	24% used drug treatment service (abstinence-based counselling, detoxification support through pharmacological intervention)	MV	*Factors associated with attending a drug treatment service*: using a new needle with each injection AOR 1.91 (95% CI 1.12–3.26); prior incarceration AOR 1.81 (95% CI 1.04–3.13)
Todd, 2011 [57]	53.8% using harm reduction services at time of enrollment 51.3% receiving needles/syringes from NSP	MV	*Factors associated with use of needle-syringe programmes*: initiated drug use with injecting AOR 2.58 (95%CI 1.22–5.44); shared injecting works in last 3 months AOR 1.79 (95% CI 1.16–2.77); prior incarceration AOR 1.57 (95%CI 1.06- 2.32); frequency of daily injection AOR 1.40 (95%CI 1.08–1.82); lived outside Afghanistan in the last 5 years AOR 0.61 (95%CI 0.41–0.91); perceived need for addiction treatment AOR 0.15 (95%CI 0.03–0.70)
Ruisenor-Escudero, 2015 [45]	54.2% retained in OST after 18 months	UV	*Factors associated with retention in OST*: being older (*p* = 0.01); being single (*p* < 0.01); reporting daily family contact in the past month (*p* < 0.01); older age at first heroin use (*p* = 0.01); fewer number of psychotic symptoms (*p* < 0.01)
Rasekh B, 2018 [43]	Completion of drug addiction treatment (psychological support, behavioural counselling and social support) (% completed n/a)	MV	*Factor associated with treatment completion*: previous history of drug addiction treatment AOR 2.46 (95%CI 1.14–5.30); attending motivational interviewing prior to hospitalization AOR 43.98 (95%CI 17.21–112.39); using heroin AOR 4.74 (95%CI 1.32–16.97)
*Colombia*			
Borda, 2021 [38]	HIV—4.7% HCV—22.8% 87.0%) HIV testing:87% HCV testing: 72.8% HIV treatment: 75% (6/8) HCV treatment:15.4% (6/39)	UV	*Factors associated with not HIV testing*: didn't know where to get a test (OR 5.68 95% CI 1.11–29.06, *p* 0.020) *Factors association with not HCV testing*: I don't want to know results (OR 2.50, 95% CI 1.13–5.52, *p* 0.021); I don't know where to go for testing (OR 7.24, 95% CI 3.10–16.91, *p* < .0001); I don't like people at testing site (OR 3.76, 95%CI 1.08–13.18, *p* 0.028) *Factors associated with not accessing HIV medical treatment*: don't have transportation to medical care (OR 0.23, 95% CI 0.05–0.99, *p* 0.034) *Factors associated with not accessing HCV medical treatment*: treated poorly at a clinic in the past (OR 3.28, 95% CI 1.09–9.89, *p* 0.027); don't trust doctors (OR 3.04, 95% 1.01–9.17, *p* 0.041); "too administrative steps" (OR 2.60, 95% 1.25–5.42, *p* 0.009); "doctor's appointment is too far" (OR 3.96, 95% I 1.87–8.40, *p* < .0001)
*Myanmar*			
Saw, 2013 [50]	77% ever tested for HIV (PWID); 46% ever tested for HIV (PWUD)	MV	*Factors associated with HIV testing among PWID*: being married AOR 0.24 (95% CI 0.06–0.94); injecting drugs at least twice daily AOR 0.30 (95% CI 0.09–0.97);.regular job AOR 4.50 (95% CI 1.08–23.17); receiving drug treatment AOR 13.07 (95% CI 3.38–50.53); self-perceiving as at risk of contracting HIV AOR 5.70 (95% CI 1.40–23.25); Shan AOR 0.30 (95%CI 0.11–0.84) or Kachin ethnicity AOR 0.30 (95%CI 0.10–0.87) compared to Burma ethnicity; reporting poly drug use within last 3 months (AOR 0.33 (95%CI 0.14–0.77); ever received drug treatment AOR 3.58 (95%CI 1.38–9.24); ever registered as a drug user AOR 4.38 (95%CI 1.31–14.65); self-perceiving as at risk of HIV infection AOR 4.46 (95%CI 2.06–9.65)
Saw, 2014 [47]	14.7% ever tested for HIV	MV	*Factors associated with HIV testing*: Female (compared to male) MA user AOR 27.02 (95%CI 11.44–63.83); having higher education AOR 3.96 (95%CI 1.20–13.02); currently living with one’s spouse/sexual partner AOR 3.92 (95%CI 1.06–14.56); being employed AOR 2.80 (95%CI 1.17–06.72); having ever visited NGO clinics or met NGO workers AOR 16.95 (95%CI 7.71–37.26); having ever been diagnosed with an STI AOR 4.83 (95%CI 2.31–10.13); having ever wanted to receive help to stop drug use AOR 4.96 (95%CI 2.23–11.05)
Aye, 2018 [30]	76% retention in OST at 6 months 90% HIV/HBV/HCV testing uptake	MV	*Factors associated with non attendance at OST (loss to follow-up)*: using drugs for experimental reasons (AOR 2.0; 95%CI 1.3–3.0); administering drugs using routes other than intravenous (e.g. inhalation) AOR 2.3 (95% CI 1.1–4.6); history of needle sharing AOR 1.5 (95% CI 1.1–2.0)
Lum, 2020 [39]	HIV testing: 45% (2016; 85% (2018); HIV: 37% (2016); 38% (2018); and initiation onto ARV: 48% (11/23 in 2016); 19% (9/47 in 2018)	UV	*Factors associated with ART initiation*: only age measures, but no significant association
O’Keefe, 2018 [42]	19% insufficient coverage of NSP (defined as numbers of n/s syrige received divided by numbers of time injecting)	MV	*Factors associated with insufficient coverage*: Mandalay sites and Pyin Oo Lwin site (compared to the Yangon sites) AOR: 0.30 (95% CIs 0.11- 0.80), AOR 0.39 (95%CIs 0.18–0.87); acquiring sterile syringes from sources other than the DIC (e.g., pharmacies or shooting galleries) AOR 2.04 (95% Cis 1.08–3.82); re-use of own unsterile syringes AOR 5.19 (95%Cis 2.57–10.48)

*MV* Multivariable; *UV* Univariable; *AHR* Adjusted hazard ratio; *AOR* adjusted odd ratio; *OR* Odds ratio

Individual-level determinants of attendance at drug treatment services included use of a new needle/syringe with each injection, use of heroin, prior attendance and use of motivational interviewing [43, 53]. Sharing injecting paraphernalia and daily injection was associated with use of needle/syringe programmes in Afghanistan, while reuse of own needles/syringes and acquiring needles/syringes from sources other than a drop in centre were associated with insufficient coverage at a needle/syringe programme in Myanmar. [42, 57] In Afghanistan fewer mental health issues, older age and family contact were associated with retention into opioid substitution therapy, while in Myanmar loss to follow-up at an opioid substitution therapy clinic was associated with using drugs through inhalation, needle/syringe sharing and using drugs for experimental purposes [30, 45]. In relation to HIV testing, being married, less frequent injecting, not engaging in poly-drug use were associated with seeking HIV testing among PWID in Lashio, Myanmar, while being female, completing higher education, living with a sex partner, using methamphetamines or having an STI was associated with increased testing among PWUD in Muse, Myanmar [47, 50]. In Colombia lack of awareness of testing sites and not wanting to know results were barriers to HIV and HCV testing [38].

Structural-level determinants of access to harm reduction services and HIV testing included living in a city, experience of living abroad, imprisonment and ethnicity. PWID in Yangon were more likely to report insufficient coverage of needle and syringe programmes than those in Mandalay (AOR 0.30 95% CIs 0.11–0.80) or Pyin Oo Lwin (AOR 0.39 95% CIs 0.18–0.87) [42]. Another study found that being of Shan (AOR 0.30 95% CI 0.11–0.84) or Kachin ethnicity (AOR 0.30 95% CI 0.10–0.87) compared to Burma ethnicity was associated with reduced odds of ever having been tested for HIV among PWUD in Lashio. [50] Living outside Afghanistan in the last five years was associated with reduced odds of using harm reduction programmes (AOR 0.61 95%CI 0.41–0.91) [57]. Two studies in Kabul found that prior incarceration was associated with greater likelihood of using harm reduction services (AOR 1.57 95% CI 1.06–2.32) or drug treatment services (AOR 1.81 95% CI 1.04–3.13) [53, 57]. Barriers to HIV treatment among PWID in Colombia include lack of transportation (OR 0.23 95% CI 0.05–0.99, *p* 0.034) [38]. In the same study past poor treatment at a clinic or lack of trust in doctor were associated barriers to HCV testing ﻿﻿﻿[38].

## Discussion

This is the first review to systematically examine the public health evidence on the health of PWUD in countries affected by armed conflict and drug production. The evidence on drug use was predominantly related to heroin injection in all three countries, but also with methamphetamine use in Myanmar and cocaine and cocaine-based derivatives in Colombia. The evidence suggests that HIV prevalence among PWID is between 2 and 8% in Afghanistan, 15–27% in Myanmar, and 2.6–6.5% in Colombia. For HCV, the prevalence among PWID ranged between 36 and 40% in Afghanistan, 48% and 76% in Myanmar, and 17.5% and 35% in Colombia. Our review highlights critical evidence gaps on health in relation to mental health and violence, with evidence on health of women who use drugs particularly lacking. We also note an absence of research on structural determinants of health among PWUD including in relation to rural contexts, drug policy and its enforcement on the ground, armed conflict, migration and forced displacement, the specific drug-producing context and increased availability of drugs. Overall, epidemiological evidence is limited, particularly in Colombia, reliant on cross-sectional data, and with available longitudinal data generally of low quality.

Prevalence of HIV and HCV are broadly comparable with global prevalence estimates among PWID of 18% for HIV and 52% for HCV [5]. Studies we identified in Colombia and Afghanistan were predominantly focussed in urban areas, and findings point to geographic differences in prevalence. Findings suggest elevated prevalence of HIV and HCV in certain cities in Afghanistan and rural border areas in Myanmar [46, 51, 63]. In Herat in Afghanistan higher prevalence is attributed by authors to the presence of a large population of repatriated Afghan refugees, particularly those who had been incarcerated, as well as high mobility and proximity of the city to drug trafficking routes [41]. These factors have been identified previously to be associated with elevated risk of HIV infection [10, 41, 64]. Other evidence from Myanmar note geographical differences in HIV prevalence among PWID, with higher prevalence in rural areas such as Bhamo and Waingmaw (61–56%) [15]. Evidence identified in our review pointed to an association between living in a rural area and elevated odds of HIV infection or working as a farmer and increased sharing of drug injecting equipment respectively [15, 40, 51]. These associations may reflect higher levels of drug use in rural farming areas where heroin is easily available and part of the economy. Other evidence identified also points to the integration of drugs in local economies, with the exchange of sex in return for amphetamines among men using drugs in Shan State, Myanmar [49]. Other factors could also explain elevated injecting and sharing of needles/syringes resulting in higher HIV prevalence in these areas. Despite widespread scale-up of services over the last ten years in Myanmar, it is widely recognised that access to effective harm reduction services in rural locations remain insufficient, creating conditions for rapid rise in HIV and HCV. [15] Low-incomes were reported universally across the studies, and injecting and unsafe practices is driven by poverty [65]. Only one study reported on experience of prison (in Afghanistan), and no studies looked at effect of policing practices or police violence, factors evidenced to be associated with elevated risk of HIV acquisition [10]. There is a need for further research to understand geographical variation in HIV prevalence and how structural factors, including the rural context and the place of drugs in economic activities, might affect risk of HIV acquisition.

Findings suggest considerable mobility among PWUD, with a high prevalence of internal migration reported in Myanmar and Afghanistan and periods of migration abroad in Afghanistan [41, 43, 44, 46, 53–59]. Findings indicate heightened vulnerability among migrants, with high-risk sexual behaviours (condomless sex, multiple partners, past STI) associated with migration status among women using drugs in Muse, Myanmar [48]. This finding is consistent with reports of high rates of drug use and sex work among the migrant workers who moved to Kachin and Shan states to work in the jade and amber mines [66]. Displacement from Afghanistan was linked to injecting drugs and reduced access to needle/syringe programmes, while initiation into injecting abroad (when displaced as refugees) was associated with increased risk of HCV infection [44, 57]. In Colombia there are an estimated 8 million IDPs [67], but no included studies reported on any aspect of migration. Further research into the role of migration and forced displacement on the health status of PWUD is needed, particularly in Colombia, but findings build on the emerging evidence base supporting the need for drug treatment services for economic or forced migrants [26, 27]. There was little information around ethnic identity to understand differential risk in relation to ethnic groups. Studies from Shan State in Myanmar and three undisclosed locations in Afghanistan suggested ethnic diversity among samples [29, 47–52]. Identifying as Pashtun was associated with elevated odds of human rights violations among women who use drugs in Afghanistan, attributed by the authors to the dominance of Taliban rules in the areas the study was conducted in and given that the Taliban are predominantly of Pashtun ethnicity [29]. People identifying as Kachin or Shan ethnicities had reduced access to HIV testing compared to the dominant Burmese ethnicity in Myanmar [50]. Further research into how ethnic identity affects risk of HIV infection and access to services is important, given the potential role of ethnic tensions in armed conflict in these contexts.

The majority of studies focused on HIV, hepatitis and sexually transmitted infections and only one study investigated mental health outcomes [29]. There is a need for further research to address gaps in evidence on mental health conditions, violence and human rights violations and how they interplay, to inform appropriate interventions. Findings also highlight the lack of epidemiological research among women who use drugs across the three countries. Structural issues relating to gender inequalities and stigma around drug use can partly explain the low representation of women in studies [68]. Some studies recruited participants from public sites where drug users were known to congregate. Evidence shows that women are more likely to use drugs at home or in private settings reducing access to both research and harm reduction services [69, 70]. International evidence points to the gender-based differences in drug use between men and women in relation to increased sexual violence, engagement in sex work, increased risk of STIs and reduced access to drug treatment services [69, 71, 72]. Ascertaining the gender-specific differences in drug-using behaviours and drug-related harms in Afghanistan, Colombia and Myanmar is a priority for further epidemiological research.

### Limitations

The broad nature of this review resulted in a diverse range of outcomes as well as exposures, and so meta-analysis was not possible. Our search focussed on PWUD not necessarily recruited in the studies during periods of armed-conflict or among those directly affected by conflict. We only identified one study that measured the effect of periods of conflict and displacement [58]. Limiting the review to papers published after 2000 may have missed papers during earlier periods of conflict. The relationship between conflict, health and drug use is complex and measuring the impact of conflict on drug use and health using quantitative methods is understandably challenging. Qualitative studies could have been included to further explore this relationship. We included only English and Spanish language studies, precluding Burmese or Afghan-language evidence and so may have missed key studies not published in English. In addition, the focus on three key countries may omit important evidence from other drug producing countries that are conflict-affected.

## Conclusions

Populations were characterized by high levels of poverty, illiteracy and unemployment, internal and external migration and imprisonment, particularly in Afghanistan and Myanmar. These structural determinants are in turn linked to elevated drug use and sexual risk behaviours, as well as HIV and HCV infection. More research is needed to understand the impact of armed-conflict and drug production on the health of PWUD, particularly in Myanmar and Colombia, to inform sustainable solutions. Epidemiological research needs to focus particularly on mental health and violence, poly drug use, particularly given the availability of cocaine-based derivatives (in Colombia) and amphetamine type stimulants (in Myanmar) [33, 34, 37, 38, 47–50, 60] The links between violence, mental health and ATS use is well established but more understanding is needed in these contexts of heightened availability and conflict, including in relation to gender differences [5, 73]. Research and services need to address intersectional vulnerabilities in relation to gender and sex work. Harm reduction services to address high-risk stimulant behaviours including injecting and smoking are urgently required. These could include the distribution of clean pipes to reduce sharing, provision of substitute drugs including coca or pharmacological substitutes such as slow-release oral amphetamines [23]. The immediate scale-up of harm reduction services to minimise injecting risks related to heroin as well as amphetamines is imperative to minimise transmission of HIV/HCV and address the multiple and linked health and social care needs that PWUD may face.

## Supplementary Information

**Additional file 1. Appendix 1**. Search strategies for Medline, Embase, PsychInfo and Global Health. **Appendix 2**. Quality Assessment.

## Data Availability

All data generated during and/or analysed during the current study are included in this published article and its Additional file 1.
