# Correction to: Determinants of health among people who use illicit drugs in the conflict-affected countries of Afghanistan, Colombia and Myanmar: a systematic review of epidemiological evidence

**DOI:** 10.1186/s13031-023-00506-z

**Published:** 2023-03-02

**Authors:** Sally O’Brien, Khine Wut Yee Kyaw, Margarita Marin Jaramillo, Bayard Roberts, Murdo Bijl, Lucy Platt

**Affiliations:** 1 Faculty of Public Health and Policy, London School of Hygiene and Tropical Medicine, London, UK; 2 Observatorio de Restitución Y Regulación de los Derechos de Propiedad Agraria, Universidad Nacional de Colombia, Bogotá, Colombia; 3 Asian Harm Reduction Network (AHRN), Yangon, Myanmar

**Correction to: Conflict and Health 16:39 (2022)** https://doi.org/10.1186/s13031-022-00467-9

The original publication of this article did not list the following authors:

- Murdo Bijl
- Khine Wut Yee Kyaw

The article has been updated to list the contributions of these authors.

