# Supplementary material for: Determinants of health among people who use illicit drugs in the conflict-affected countries of Afghanistan, Colombia and Myanmar: a systematic review of epidemiological evidence
Source: Confl Health. 2022 Jul 7;16:39. doi: 10.1186/s13031-022-00467-9 (PMC9264525; doi:10.1186/s13031-022-00467-9)
Supplement: Supplementary file 1 — Additional file 1. Appendix 1. Search strategies for Medline, Embase, PsychInfo and Global Health. Appendix 2. Quality Assessment. [file 13031_2022_467_MOESM1_ESM.docx]

# **Appendix 1: Search strategies for Medline, Embase, PsychInfo and Global Health**

Medline

| **#** | **Searches** | **Results** |
| --- | --- | --- |

| 1 | Burma.mp. or Myanmar/ | 3411 |
| --- | --- | --- |
| 2 | Myanmar.mp. or Myanmar/ | 4900 |
| 3 | Burmese/ | 70193 |
| 4 | Colombia.mp. or Colombia/ | 17518 |
| 5 | Colombian.mp. or Colombian/ | 5719 |
| 6 | Afghanistan.mp. or Afghanistan/ | 7488 |
| 7 | Afghan.mp. or Afghan/ | 3971 |
| 8 | 1 or 2 or 3 or 4 or 5 or 6 or 7 | 103153 |
| 9 | exp substance abuse/ or exp "substance use"/ | 294006 |
| 10 | exp drug abuse/ | 294006 |
| 11 | exp drug dependence/ | 294006 |
| 12 | exp cocaine/ | 26182 |
| 13 | exp amphetamine abuse/ or exp amphetamine/ or exp amphetamine dependence/ | 22269 |
| 14 | exp "cannabis use"/ or exp cannabis addiction/ or exp cannabis/ | 10857 |
| 15 | exp opiate/ | 89694 |
| 16 | exp diamorphine/ | 5943 |
| 17 | exp psychedelic agent/ | 26142 |
| 18 | ("substance use" or "substance abuse" or "drug use" or "inject* drug use" or addict*).mp. [mp=title, abstract, original title, name of substance word, subject heading word, floating sub-heading word, keyword heading word, organism supplementary concept word, protocol supplementary concept word, rare disease supplementary concept word, unique identifier, synonyms] | 188044 |
| 19 | ("substance-related disorder" or cocaine or amphetamine or methamphetamine or khat or qat).mp. [mp=title, abstract, original title, name of substance word, subject heading word, floating sub-heading word, keyword heading word, organism supplementary concept word, protocol supplementary concept word, rare disease supplementary concept word, unique identifier, synonyms] | 79200 |
| 20 | (cannabis or marijuana or opiate or opium or heroin or "party drugs" or "club drugs").mp. [mp=title, abstract, original title, name of substance word, subject heading word, floating sub-heading word, keyword heading word, organism supplementary concept word, protocol supplementary concept word, rare disease supplementary concept word, unique identifier, synonyms] | 80114 |
| 21 | ("bath salts" or ecstasy or MDMA or ketamine or hallucinogens or inhalants).mp. [mp=title, abstract, original title, name of substance word, subject heading word, floating sub-heading word, keyword heading word, organism supplementary concept word, protocol supplementary concept word, rare disease supplementary concept word, unique identifier, synonyms] | 34360 |
| 22 | exp intravenous drug abuse/ | 16066 |
| 23 | 9 or 10 or 11 or 12 or 13 or 14 or 15 or 16 or 17 or 18 or 19 or 20 or 21 or 22 | 557473 |
| 24 | exp health care availability/ or exp reproductive health/ or exp health survey/ or exp health care need/ or exp health care access/ or exp sexual health/ or exp health care/ or exp health status/ or exp health care delivery/ or exp health/ or exp mental health/ | 2226510 |
| 25 | exp harm reduction/ or exp methadone/ | 16512 |
| 26 | exp drug dependence treatment/ | 0 |
| 27 | (HIV or hepatitis or HBV or HCV or sexually transmitted or chlamydia or gonorrhoea or syphilis or Treponema Pallidum or trichomonas or pelvic inflammatory disease or AIDS or sexual health or tuberculosis or TB).mp. [mp=title, abstract, original title, name of substance word, subject heading word, floating sub-heading word, keyword heading word, organism supplementary concept word, protocol supplementary concept word, rare disease supplementary concept word, unique identifier, synonyms] | 1068132 |
| 28 | (Morbidity or mortality or cirrhosis or cancer or death or overdose or suicide or suicidal or depression or anxiety or psychosis).mp. [mp=title, abstract, original title, name of substance word, subject heading word, floating sub-heading word, keyword heading word, organism supplementary concept word, protocol supplementary concept word, rare disease supplementary concept word, unique identifier, synonyms] | 4394489 |
| 29 | (violen* or brutal* or attack* or assault* or rape* or safety or security or protection or murder or homicide).mp. [mp=title, abstract, original title, name of substance word, subject heading word, floating sub-heading word, keyword heading word, organism supplementary concept word, protocol supplementary concept word, rare disease supplementary concept word, unique identifier, synonyms] | 1286077 |
| 30 | (clinic* or outreach or hospital* or health care or health service* or psychologist* or psychiatrist* or counselling or needle* or syringe or harm reduction or methadone or buprenorphine or opioid substitution or nalaxone or psychosocial support or housing or accommodation or shelter* or social services or social welfare or social security or primary care or GP or general practi* or family doctor* or dentist* or dental care or dental treatment or test* or treat* or specialist service or Social care service* or social care* or voluntary sector* or voluntary service* or voluntary care service* or community care* or social service or safeguarding or child protection or evict* or loans or access to clean water).mp. [mp=title, abstract, original title, name of substance word, subject heading word, floating sub-heading word, keyword heading word, organism supplementary concept word, protocol supplementary concept word, rare disease supplementary concept word, unique identifier, synonyms] | 13876427 |
| 31 | 24 or 25 or 26 or 27 or 28 or 29 or 30 | 16782927 |
| 32 | 8 and 23 and 31 | 1498 |
| 33 | limit 32 to yr="2000 -Current" | 1365 |
| 34 | limit 32 to (human and yr="2000 -Current") | 1238 |

**Embase**

| **#** | **Searches** | **Results** |
| --- | --- | --- |
| 1 | myanmar.mp. or exp Myanmar/ | 4192 |
| 2 | burma.mp. or exp Burma/ | 3660 |
| 3 | exp burmese/ | 113 |
| 4 | colombia.mp. or exp Colombia/ | 20116 |
| 5 | colombian.mp. or exp Colombian/ | 6190 |
| 6 | Afghanistan.mp. or exp Afghanistan/ | 8087 |
| 7 | exp Afghan/ or afghan*.mp. | 8614 |
| 8 | 1 or 2 or 3 or 4 or 5 or 6 or 7 | 35055 |
| 9 | exp substance abuse/ or exp "substance use"/ | 502526 |
| 10 | exp drug abuse/ | 107991 |
| 11 | exp drug dependence/ | 205750 |
| 12 | exp cocaine/ | 50594 |
| 13 | exp amphetamine abuse/ or exp amphetamine/ or exp amphetamine dependence/ | 26289 |
| 14 | exp "cannabis use"/ or exp cannabis addiction/ or exp cannabis/ | 38963 |
| 15 | exp opiate/ | 77102 |
| 16 | exp diamorphine/ | 20852 |
| 17 | exp psychedelic agent/ | 67810 |
| 18 | ("substance use" or "substance abuse" or "drug use" or "inject* drug use" or addict*).mp. [mp=title, abstract, heading word, drug trade name, original title, device manufacturer, drug manufacturer, device trade name, keyword, floating subheading word, candidate term word] | 331728 |
| 19 | ("substance-related disorder" or cocaine or amphetamine or methamphetamine or khat or qat).mp. [mp=title, abstract, heading word, drug trade name, original title, device manufacturer, drug manufacturer, device trade name, keyword, floating subheading word, candidate term word] | 102371 |
| 20 | (cannabis or marijuana or opiate or opium or heroin or "party drugs" or "club drugs").mp. [mp=title, abstract, heading word, drug trade name, original title, device manufacturer, drug manufacturer, device trade name, keyword, floating subheading word, candidate term word] | 192054 |
| 21 | ("bath salts" or ecstasy or MDMA or ketamine or hallucinogens or inhalants).mp. [mp=title, abstract, heading word, drug trade name, original title, device manufacturer, drug manufacturer, device trade name, keyword, floating subheading word, candidate term word] | 47647 |
| 22 | exp intravenous drug abuse/ | 10138 |
| 23 | 9 or 10 or 11 or 12 or 13 or 14 or 15 or 16 or 17 or 18 or 19 or 20 or 21 or 22 | 1095681 |
| 24 | exp health care availability/ or exp reproductive health/ or exp health survey/ or exp health care need/ or exp health care access/ or exp sexual health/ or exp health care/ or exp health status/ or exp health care delivery/ or exp health/ or exp mental health/ | 5279191 |
| 25 | exp harm reduction/ or exp methadone/ | 33150 |
| 26 | exp drug dependence treatment/ | 21219 |
| 27 | (HIV or hepatitis or HBV or HCV or sexually transmitted or chlamydia or gonorrhoea or syphilis or Treponema Pallidum or trichomonas or pelvic inflammatory disease or AIDS or sexual health or tuberculosis or TB).mp. [mp=title, abstract, heading word, drug trade name, original title, device manufacturer, drug manufacturer, device trade name, keyword, floating subheading word, candidate term word] | 1090648 |
| 28 | (Morbidity or mortality or cirrhosis or cancer or death or overdose or suicide or suicidal or depression or anxiety or psychosis).mp. [mp=title, abstract, heading word, drug trade name, original title, device manufacturer, drug manufacturer, device trade name, keyword, floating subheading word, candidate term word] | 6059887 |
| 29 | (violen* or brutal* or attack* or assault* or rape* or safety or security or protection or murder or homicide).mp. [mp=title, abstract, heading word, drug trade name, original title, device manufacturer, drug manufacturer, device trade name, keyword, floating subheading word, candidate term word] | 1832474 |
| 30 | (clinic* or outreach or hospital* or health care or health service* or psychologist* or psychiatrist* or counselling or needle* or syringe or harm reduction or methadone or buprenorphine or opioid substitution or nalaxone or psychosocial support or housing or accommodation or shelter* or social services or social welfare or social security or primary care or GP or general practi* or family doctor* or dentist* or dental care or dental treatment or test* or treat* or specialist service or Social care service* or social care* or voluntary sector* or voluntary service* or voluntary care service* or community care* or social service or safeguarding or child protection or evict* or loans or access to clean water).mp. [mp=title, abstract, heading word, drug trade name, original title, device manufacturer, drug manufacturer, device trade name, keyword, floating subheading word, candidate term word] | 17037964 |
| 31 | 24 or 25 or 26 or 27 or 28 or 29 or 30 | 19803354 |
| 32 | 8 and 23 and 31 | 2148 |
| 33 | limit 32 to yr="2000 -Current" | 2125 |

**PsychINFO**

| **#** | **Searches** | **Results** |
| --- | --- | --- |
| 1 | myanmar.mp. | 293 |
| 2 | burma.mp. | 244 |
| 3 | burmese.mp. | 222 |
| 4 | colombia.mp. | 2758 |
| 5 | colombian.mp. | 1407 |
| 6 | Afghanistan.mp. | 2900 |
| 7 | afghan*.mp. | 3399 |
| 8 | 1 or 2 or 3 or 4 or 5 or 6 or 7 | 7288 |
| 9 | exp Drug Addiction/ | 18694 |
| 10 | exp Drug Abuse/ | 46843 |
| 11 | exp Drug Usage/ | 93612 |
| 12 | exp Hallucinogenic Drugs/ | 3735 |
| 13 | exp "Cannabis Use Disorder"/ or exp Cannabis/ | 8164 |
| 14 | exp Amphetamine/ | 13211 |
| 15 | exp Heroin Addiction/ or exp Heroin/ | 4946 |
| 16 | ("substance use" or "substance abuse" or "drug use" or "inject* drug use" or addict*).mp. [mp=title, abstract, heading word, table of contents, key concepts, original title, tests & measures, mesh] | 138792 |
| 17 | ("substance-related disorder" or cocaine or amphetamine or methamphetamine or khat or qat).mp. [mp=title, abstract, heading word, table of contents, key concepts, original title, tests & measures, mesh] | 36084 |
| 18 | (cannabis or marijuana or opiate or opium or heroin or "party drugs" or "club drugs").mp. [mp=title, abstract, heading word, table of contents, key concepts, original title, tests & measures, mesh] | 34393 |
| 19 | ("bath salts" or ecstasy or MDMA or ketamine or hallucinogens or inhalants).mp. [mp=title, abstract, heading word, table of contents, key concepts, original title, tests & measures, mesh] | 9368 |
| 20 | 9 or 10 or 11 or 12 or 13 or 14 or 15 or 16 or 17 or 18 or 19 | 248453 |
| 21 | exp Health Care Seeking Behavior/ or exp Mental Health/ or exp Health Care Services/ or exp Health Outcomes/ or exp Physical Health/ or exp Health/ or exp Mental Health Services/ or exp Health Service Needs/ or exp Health Status/ or exp Public Health/ or exp Reproductive Health/ or exp "Health Related Quality of Life"/ or exp Sexual Health/ or exp Health Care Access/ or exp Health Care Utilization/ | 410250 |
| 22 | exp Harm Reduction/ | 3401 |
| 23 | exp Addiction Treatment/ or exp "Substance Use Treatment"/ | 32679 |
| 24 | (HIV or hepatitis or HBV or HCV or sexually transmitted or chlamydia or gonorrhoea or syphilis or Treponema Pallidum or trichomonas or pelvic inflammatory disease or AIDS or sexual health or tuberculosis or TB).mp. [mp=title, abstract, heading word, table of contents, key concepts, original title, tests & measures, mesh] | 83679 |
| 25 | (Morbidity or mortality or cirrhosis or cancer or death or overdose or suicide or suicidal or depression or anxiety or psychosis).mp. [mp=title, abstract, heading word, table of contents, key concepts, original title, tests & measures, mesh] | 661269 |
| 26 | (violen* or brutal* or attack* or assault* or rape* or safety or security or protection or murder or homicide).mp. [mp=title, abstract, heading word, table of contents, key concepts, original title, tests & measures, mesh] | 259873 |
| 27 | (clinic* or outreach or hospital* or health care or health service* or psychologist* or psychiatrist* or counselling or needle* or syringe or harm reduction or methadone or buprenorphine or opioid substitution or nalaxone or psychosocial support or housing or accommodation or shelter* or social services or social welfare or social security or primary care or GP or general practi* or family doctor* or dentist* or dental care or dental treatment or test* or treat* or specialist service or Social care service* or social care* or voluntary sector* or voluntary service* or voluntary care service* or community care* or social service or safeguarding or child protection or evict* or loans or access to clean water).mp. [mp=title, abstract, heading word, table of contents, key concepts, original title, tests & measures, mesh] | 2120050 |
| 28 | 21 or 22 or 23 or 24 or 25 or 26 or 27 | 2554235 |
| 29 | 8 and 20 and 28 | 506 |
| 30 | limit 29 to yr="2000 -Current" | 418 |

**Global Health**

| **#** | **Searches** | **Results** |
| --- | --- | --- |
| 1 | myanmar.mp. or exp Myanmar/ | 4255 |
| 2 | burma.mp. | 4073 |
| 3 | burmese.mp. | 438 |
| 4 | afghanistan.mp. or exp Afghanistan/ | 2302 |
| 5 | afghan.mp. | 403 |
| 6 | colombia.mp. or exp Colombia/ | 10726 |
| 7 | colombian.mp. | 2700 |
| 8 | 1 or 2 or 3 or 4 or 5 or 6 or 7 | 18229 |
| 9 | exp drug abuse/ | 16976 |
| 10 | exp substance abuse/ | 23650 |
| 11 | (drug addiction or drug users).sh. | 8212 |
| 12 | exp cocaine/ | 2655 |
| 13 | exp amfetamine/ | 688 |
| 14 | exp Cannabis/ | 4686 |
| 15 | exp opium/ | 542 |
| 16 | exp heroin/ | 1832 |
| 17 | ("substance use" or "substance abuse" or "drug use" or "inject* drug use" or addict*).mp. [mp=abstract, title, original title, broad terms, heading words, identifiers, cabicodes] | 39235 |
| 18 | ("substance-related disorder" or cocaine or amphetamine or methamphetamine or khat or qat).mp. [mp=abstract, title, original title, broad terms, heading words, identifiers, cabicodes] | 5992 |
| 19 | (cannabis or marijuana or opiate or opium or heroin or "party drugs" or "club drugs").mp. [mp=abstract, title, original title, broad terms, heading words, identifiers, cabicodes] | 10662 |
| 20 | ("bath salts" or ecstasy or MDMA or ketamine or hallucinogens or inhalants).mp. [mp=abstract, title, original title, broad terms, heading words, identifiers, cabicodes] | 1899 |
| 21 | 9 or 10 or 11 or 12 or 13 or 14 or 15 or 16 or 17 or 18 or 19 or 20 | 47202 |
| 22 | exp health/ or exp health services/ or exp mental health/ or exp health behaviour/ or exp health care utilization/ or exp sexual health/ or exp reproductive health/ | 337124 |
| 23 | harm reduction.mp. or risk reduction.sh. or injecting drug users.sh. or needle exchange schemes.sh. or injecting drug abuse.sh. or risk groups.sh. | 32297 |
| 24 | addiction treatment.mp. | 355 |
| 25 | (HIV or hepatitis or HBV or HCV or sexually transmitted or chlamydia or gonorrhoea or syphilis or Treponema Pallidum or trichomonas or pelvic inflammatory disease or AIDS or sexual health or tuberculosis or TB).mp. [mp=abstract, title, original title, broad terms, heading words, identifiers, cabicodes] | 371591 |
| 26 | (Morbidity or mortality or cirrhosis or cancer or death or overdose or suicide or suicidal or depression or anxiety or psychosis).mp. [mp=abstract, title, original title, broad terms, heading words, identifiers, cabicodes] | 518878 |
| 27 | (violen* or brutal* or attack* or assault* or rape* or safety or security or protection or murder or homicide).mp. [mp=abstract, title, original title, broad terms, heading words, identifiers, cabicodes] | 336309 |
| 28 | (clinic* or outreach or hospital* or health care or health service* or psychologist* or psychiatrist* or counselling or needle* or syringe or harm reduction or methadone or buprenorphine or opioid substitution or nalaxone or psychosocial support or housing or accommodation or shelter* or social services or social welfare or social security or primary care or GP or general practi* or family doctor* or dentist* or dental care or dental treatment or test* or treat* or specialist service or Social care service* or social care* or voluntary sector* or voluntary service* or voluntary care service* or community care* or social service or safeguarding or child protection or evict* or loans or access to clean water).mp. [mp=abstract, title, original title, broad terms, heading words, identifiers, cabicodes] | 1979059 |
| 29 | 22 or 23 or 24 or 25 or 26 or 27 or 28 | 2483749 |
| 30 | 8 and 21 and 29 | 345 |
| 31 | limit 30 to yr="2000 -Current" | 304 |

# **Appendix 2 Quality Assessment**

| **Newcastle-Ottowa Quality Assessment Scale (Cross- sectional studies)** | | | | | | | | |
| --- | --- | --- | --- | --- | --- | --- | --- | --- |
|  | **Representativeness of sample *** | **Sample size*** | **Non-respondents*** | **Ascertainment of the Exposure**** | **Confounding factors controlled**** | **Assessment of the outcome**** | **Statistical test*** | **Total (max 10)** |
| Abadi, 2012 |  |  |  | * |  | * |  | 2 |
| Bautista, 2010 | * |  |  | * | * | ** | * | 6 |
| Berbesi, Fernandez, 2013 (JSU) | * | * |  | * | * | * | * | 6 |
| Berbesi, Fernandez, 2013 (SM) | * |  |  | * |  | * |  | 3 |
| Berbesi, Fernandez, 2015 | * | * |  | * | * | * | * | 6 |
| Berbesi, Fernandez, 2017 | * | * |  | * | * | * | * | 6 |
| Berbesi, Fernandez, 2017 | * | * |  | * | * | ** | * | 7 |
| Berbesi, 2020 | * | * |  | * | * | ** | * | 7 |
| Borda, 2021 |  |  |  | * |  | * | * | 3 |
| Morineau, 2000 |  |  |  | * |  | * | * | 2 |
| Nassir, 2011 | * |  |  | * | ** | ** | * | 7 |
| O’Keefe, 2010 | * |  |  | * | ** | ** | * | 7 |
| Rasekh, 2018 | ** |  |  | * | ** | * | * | 7 |
| Rasekh, 2019 | * | * |  | * | * | * | * | 6 |
| Ruisenor-Escudero, 2014 | ** | * |  | * | ** | ** | * | 9 |
| Ruisenor-Escudero, 2015 | * |  |  | * |  | * | * | 4 |
| Saw, 2013 | ** |  |  | * | ** | * | * | 7 |
| Saw, 2014 | ** |  |  | * | ** | * | * | 7 |
| Saw, 2018 | ** |  |  | * | ** | * | * | 7 |
| Saw, 2016 | ** |  |  | ** | ** | * | * | 8 |
| Swe, 2012 |  |  |  | * |  |  | * | 2 |
| Todd, 2007a | * | * |  | * | * | ** | * | 7 |
| Todd, 2007 |  | * |  | * | * | * | * | 5 |
| Todd, 2010 | * |  |  | ** | ** | ** | * | 8 |
| Todd, 2009 | * |  |  | * | * | * | * | 5 |
| Todd, 2011 | * | * |  | * | * | * | * | 6 |
| Toro-Tobon, 2018 | * | * |  | * | * | ** | * | 7 |
| Toro-Tobon, 2020 | * | * |  | * | * | ** | * | 7 |
| Zafar, 2003 |  |  |  |  | ** | * | * | 4 |
| Zhou, 2011 | * |  |  |  |  | ** | * | 4 |

| **Newcastle-Ottowa Quality Assessment Scale (Case-control studies)** | | | | | | | | | |
| --- | --- | --- | --- | --- | --- | --- | --- | --- | --- |
|  | **Is the case definition adequate*** | **Representativeness of cases*** | **Selection of controls*** | **Definition of controls*** | **Comparability of cases and control **** | **Ascertainment of exposure*** | **Same method od ascertainment for both*** | **Non-response rate*** | **Total (maximum score is 9)** |
| Swe, 2010 | * | * | * | * |  | * | * | * | 7 |

| **Newcastle-Ottawa Scale (Cohort studies)** | | | | | | | | | |
| --- | --- | --- | --- | --- | --- | --- | --- | --- | --- |
|  | **Representativeness of exposed** | **Selection of non-exposed** | **Ascertainment of exposure** | **Outcome not present at start** | **Confounding factors controlled**** | **Assessment of outcome** | **Follow-up long enough** | **Adequacy of follow-up** | **Total (max 9)** |
| Aye, 2018 | * |  | * | * | ** | * | * | * | 8 |
| Todd, 2016 | * | * |  |  | ** |  | * |  | 5 |
| Todd, 2015 | * | * |  | * | ** | * | * |  | 7 |
| Lum, 2021 | * | * | * | * |  | * | * |  | 6 |
